# The CarerQol Instrument: A Systematic Review, Validity Analysis, and Generalization Reliability Study

**DOI:** 10.3390/jcm14061916

**Published:** 2025-03-12

**Authors:** Elena Cejalvo, Manuel Martí-Vilar, Júlia Gisbert-Pérez, Laura Badenes-Ribera

**Affiliations:** 1 Department of Basic Psychology, Faculty of Psychology and Speech Therapy, University of Valencia, 46010 Valencia, Spain; ecehe@alumni.uv.es; 2 Department of Behavioural Sciences Methodology, Faculty of Psychology and Speech Therapy, University of Valencia, 46010 Valencia, Spain; julia.gisbert@uv.es (J.G.-P.); laura.badenes@uv.es (L.B.-R.)

**Keywords:** informal caregivers, reliability, validity, meta-analysis, systematic review

## Abstract

**Background/Objectives**: The CarerQol instrument is used to measure the quality of life of informal caregivers and to assess the impact that caring for a dependent person has on them. The scale consists of two parts, CarerQol-7D, which measures the effects of informal care using two positive and five negative domains, and CarerQol-VAS, which measures happiness on a visual analog scale. **Methods**: In the present work, we conducted a systematic review of the instrument since its development in 2006, followed by a (convergent, clinical, and discriminative) validity analysis and a meta-analysis of the reliability of generalizing CarerQol. A total of 54 articles that used CarerQol were identified. **Results**: The instrument was found to have good convergent, clinical, and discriminant validity, although the average reliability coefficient was 0.67 (95% CI [0.56, 0.75]) for Cronbach’s alpha coefficients and 0.62 (95% CI [0.04, 0.89]) for test–retest reliability coefficients, with a high degree of heterogeneity between the coefficients. **Conclusions**: According to the psychometric theory, CarerQol is a reliable instrument and can be used for exploratory purposes in the field of research, although it should be used with caution when making decisions in clinical practice.

## 1. Introduction

### 1.1. The Reality of Informal Care

Informal carers are crucial in the evolution of care, protecting and enriching the self-esteem of dependent people, accounting for up to 80% of long-term care in Europe [1]. However, taking on this role involves significant challenges, such as combining caregiving with personal and community obligations, which can lead to stress, social isolation, financial problems, and increased mortality risk [2,3,4,5,6,7,8]. Although social support acts as a protective factor [9], lack of training and compensation increases caregivers’ stress and vulnerability [10]. Some countries, such as Sweden, Denmark, and Australia, offer support programs and funding [11,12], but in regions with fewer resources, caregivers face greater difficulties [2,13,14]. Despite the negative aspects, informal care also has positive impacts, such as emotional well-being, personal fulfillment, and strengthened family ties [15,16]. It is therefore essential to allocate funds to support this vulnerable group and to develop tools, such as CarerQol, that assess both the burdens and positive aspects of informal care.

### 1.2. Other Instruments Measuring Caregiver Burden

A number of scales can be used to assess caregiver burden, such as the CSI instrument [17], the SCQ questionnaire, the Carer Experience Scale (CES) [18,19], or the SRB scale [20]. However, these instruments neglect the effects of informal caregiving and lack an assessment of the burden from the informal caregiver’s own experience [21]. These authors therefore believed that it was imperative to create an instrument that understood the reality of caregiving and incorporated an assessment of the effects of caregiving on informal caregivers in the form of cost and consequence analysis. For this reason, the authors considered it crucial to develop an instrument that would reflect the reality of informal caregiving and include an assessment of the effects of caregiving on the lives of informal caregivers, taking into account both costs and consequences. From this conception, the CarerQol instrument emerged, with the purpose of describing the profile of carers and assessing the impact this role has on their lives [15].

### 1.3. The Care-Related Quality of Life Instrument (CarerQol)

Brouwer and colleagues [15] took the EuroQol instrument [22] as their starting point as it contains an explanation of the health situation and a health status assessment item [23] to develop CarerQol, which consists of CarerQol-7D and CarerQol-VAS. The first part measures the caregiver’s burden and its impact on seven dimensions, five of which refer to negative aspects (problems associated with relations, mental health, reconciling care with daily activities, finances and problems related to physical condition). The remaining two dimensions refer to positive aspects (the support received and the satisfaction from caregiving).

The dimensions that make up the test are as follows: I find it satisfying to perform my care tasks, I have relational problems with the caregiver (e.g., he/she is very demanding or behaves differently; communication problems), I have problems with my own mental health (e.g., stress, fear, despondency, depression, worry about the future), I have problems combining my care tasks with my daily activities (e.g., household activities, work, studies, family and leisure activities), I have financial problems because of my care tasks, I have support for my care tasks when I need it (e.g., from relatives, friends, neighbours, acquaintances) and I have problems with my own physical health (e.g., more often ill, tiredness, physical stress). Each dimension contains one statement with a 3-choice response scale (1: Not at all; 2: Somewhat; 3: Very much). The higher the sum score, the better the situation and the better the quality of care, while low scores are associated with poor care situations with many problems in the negative dimensions, where very little support is received from others and satisfaction is almost non-existent [24].

The second part of the questionnaire assesses the satisfaction received from caregiving using a 10-point scale that ascends in proportion to the satisfaction received [15].

The instrument is currently translated into 12 other languages [25] and can be distributed to different caregiver populations as its different items do not refer to specific situations, so that it can be used to compare the effects of caregiving in different caregiver groups and in people with disabilities [26]. The instrument thus enables descriptive research on the effects of caregiving on caregivers [27].

Other instruments are also currently in use, such as ASCOT-Carer, which examines the quality of life related to social care through seven domains. However, studies such as that by Rand et al. [28] consider that this instrument does not take into account aspects that may be outside the social care setting, which could be a serious limitation when compared to CareQol.

CarerQol can be a valuable tool for health professionals, researchers, and policy makers to assess and address the needs of informal carers and develop specific interventions to improve their quality of life.

### 1.4. Objetives and Relevance of Current Study

The main objective of this study was to collect and analyze the psychometric properties of the CarerQol instrument (reliability, convergent, clinical, and discriminative validity) and its applications since its creation in 2006, as there were no previous reviews integrating these aspects. Using the PRISMA method [29], published studies were reviewed, considering design, sampling, caregiver profile, conclusions, and limitations. In addition, the informal caregiving situation was contextualized and a reliability meta-analysis was conducted using the REGEMA method [30] to assess the consistency of the instrument in different settings. The underlying hypothesis was that CarerQol is a valid and reliable tool for measuring both burdens and positive aspects of informal care, adaptable to various clinical and population contexts. In the clinical setting, the study sought to support its use in assessing the well-being of informal caregivers, highlighting its relevance for identifying needs and designing support interventions.

## 2. Materials and Methods

### 2.1. Registration of Review

The review was registered in PROSPERO (CRD42022331812), which confirmed that our study was not duplicated and was free of any reporting bias.

### 2.2. Eligibility Criteria

The criteria for including studies were as follows: (a) the research should be original; (b) be published in peer-reviewed journals between 2006 and January 2022 (both inclusive); (c) have applied the CarerQol instrument in its original structure; (d) contain seven dimensions and an analog scale that measures happiness; (e) be available in full text; and (f) be published in English or Spanish. There were no limitations on research design, geographic location, or sample type.

The meta-analysis was conducted with the same inclusion criteria as the literature review and a new criterion was added that required manuscripts to provide a reliability estimate based on the sample analyzed in the study.

### 2.3. Search Itinerary

The electronic search of the literature was carried out between September 2021 and January 2022 in different databases, followed by a manual search in which the references of the manuscripts included in the study were reviewed to identify and to include new studies that met the previously stated standards.

The electronic search was carried out in the following databases: WoS, Scopus, Pubmed, Eric, Embase, and ProQuest. The following word combinations were used: caregivers* AND CarerQol*. Therefore, words that were synonymous with the word caregivers were not used.

The “scientific journal” field was chosen to refine the search in the ProQuest database, although no term was limited in the other databases.

### 2.4. Study Selection Process

There were 406 articles identified (Pubmed: 50, WOS: 60, Scopus: 47, Eric: 7, Embase: 53, ProQuest: 190), of which 212 were removed as duplicated, leaving a total of 194 articles for review. The first step was to read the abstract, which resulted in 34 articles being eliminated for not dealing with the topic in question, leaving 160 articles that were analyzed in their entirety. After reading the texts in depth, 107 articles were eliminated, so that 54 manuscripts were finally included in the review. To ensure inter-rater reliability, this process was carried out independently by two authors using the Covidence tool.

**Figure 1:**
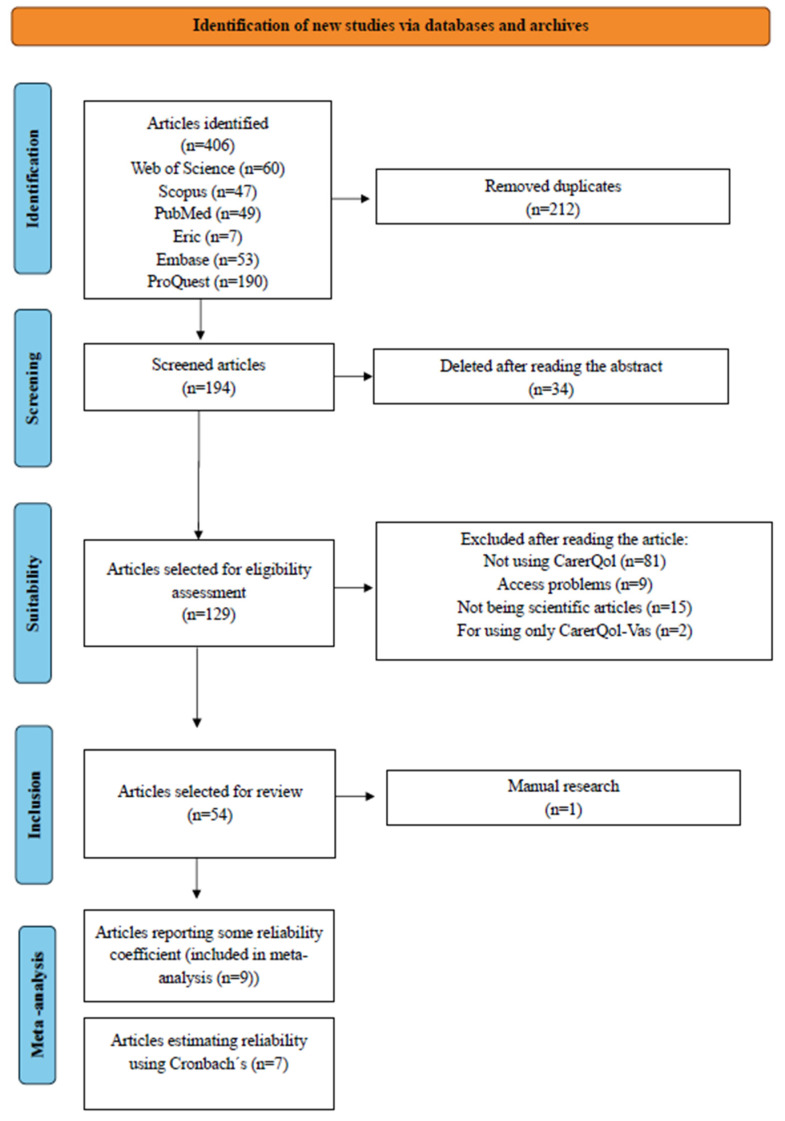
Flowchart according to PRISMA.

Only 9 of these 54 articles reported reliability estimates based on the samples analyzed in the respective studies, which meant that only these 9 studies were used for meta-analysis. Figure 1 shows the flowchart used, which describes the study selection process and relevant details.

### 2.5. Screening Conducted

The articles were analyzed independently by the three authors responsible for this work, based on the previously established inclusion criteria. Inconsistencies were resolved by consensus. Finally, the quality criteria to be used were confirmed by all three researchers.

### 2.6. The Information Extracted from the Studies

A data extraction protocol was designed and applied systematically. The following details were recorded: (a) author and year, (b) country, (c) continent, (d) type of sample, (e) collective care, (f) version of the scale, (g) type of caregiver, (h) distribution by sex, (i) age of the caregivers, (j) educational level of the caregivers, (k) variables measured in the study, (l) main results obtained, and (m) limitations reported in the studies. The reported reliability coefficients (Cronbach’s alpha and test–retest) and the data referring to the validity of the CarerQol scores were also recorded.

### 2.7. Validity Analysis Study

Table A3 (Appendix A.1) exclusively contains articles measuring the psychometric properties of the instrument. Of the 54 items included, only 12 were psychometric. The table was organized according to authorship, sample (n), gender, and the psychometric properties analyzed (convergent validity, clinical validity, and construct validity). The validity of the scores were analyzed by the classification proposed in Hoefman et al. [31], which distinguishes among the following: Convergent validity, which assesses whether the constructs underlying CarerQol are similar to other instruments that also assess caregiver burden (strong correlation indicates that CarerQol is effective in accurately measuring what it sets out to assess and that it is consistent with other measurement tools in this area). Discriminative validity, is a vital component of any measurement tool, demonstrating its capacity to effectively distinguish between different groups or conditions that are anticipated to differ in the variable being evaluated. This quality is essential for ensuring that the instrument meaningfully captures variations in the construct it aims to measure. Clinical validity refers to the extent to which a measurement instrument accurately assesses the construct it is intended to measure and demonstrates a meaningful relationship with relevant explanatory variables. These variables can include factors such as age, gender, employment status, and frequency of care.

Although the Hoefman et al. [31] classification was used to distinguish the different types of validity, the quality of measurement tests was assessed through the COSMIN guidelines [32]. The COSMIN initiative aims to improve the selection of health measurement instruments in both research and clinical practice by developing tools to select the most appropriate instrument for a given situation. These guidelines provide a comprehensive framework for assessing both the reliability and validity of instruments, ensuring that all relevant methodological aspects are considered.

### 2.8. Reliability Estimates

Two reliability estimates were considered for the meta-analytic study: Cronbach’s alpha coefficients and intraclass correlation coefficients. The former assesses the reliability of the internal consistency of the measures and the latter assesses the reliability as the temporal stability of the scores. Cronbach’s alpha coefficients were transformed by Bonett’s formula [33] and the intraclass correlation coefficients were translated to Fisher’s Z to stabilize their variance and normalize their distributions [34]. They were then back-transformed into Cronbach’s alpha coefficients and intraclass correlation coefficient metrics to facilitate interpreting the results of the meta-analysis. In the statistical analysis, a random effects model was applied to estimate the mean reliability coefficient and its 95% confidence interval using the improved method proposed by Hartung and Knapp [35].

### 2.9. Statistical Analysis

Two separate meta-analyses were performed for Cronbach’s Alpha coefficients (internal consistency) and intraclass correlation coefficients (test–retest reliability). In both cases, a random effects model was assumed to compute the summary statistics of the reliability coefficient using the improved procedure proposed by Hartung and Knapp [35]. Forest plots were displayed to show the average and individual reliability estimate, with 95% CI, and visually assess the heterogeneity of the reliability coefficients in the meta-analyses. The *Q*-statistic, completed with the *I*^2^ index, was computed to examine the variability among the reliability estimates. A *Q*-statistic with a value *p* ≤ 0.05 was considered to indicate variability. *I*^2^ index values around 25% can be considered as representative of low heterogeneity, 50% as moderate, and 75% as high heterogeneity [36]. The sample characteristics of the studies inducing and reporting test reliability were compared to analyze the degree to which the meta-analysis results can be generalized to all the studies that applied CarerQol. The statistical analyses were conducted in R using the Metafor Package [37].

## 3. Results

The results section includes an analysis of the methodological aspects of the studies reviewed, focusing on study design, sample size, and data collection methods. It also examines the characteristics of caregiver samples, including age, gender, education, and ethnicity. Additionally, it explores the psychometric properties of the CarerQOL instrument, specifically in validation studies, feasibility and reliability assessments, and valuation studies.

Table A1 (Appendix A.1) lists the main results and characteristics of the 54 studies analyzed, and out of these 12, evaluated the psychometric properties of the instrument.

**Figure 2:**
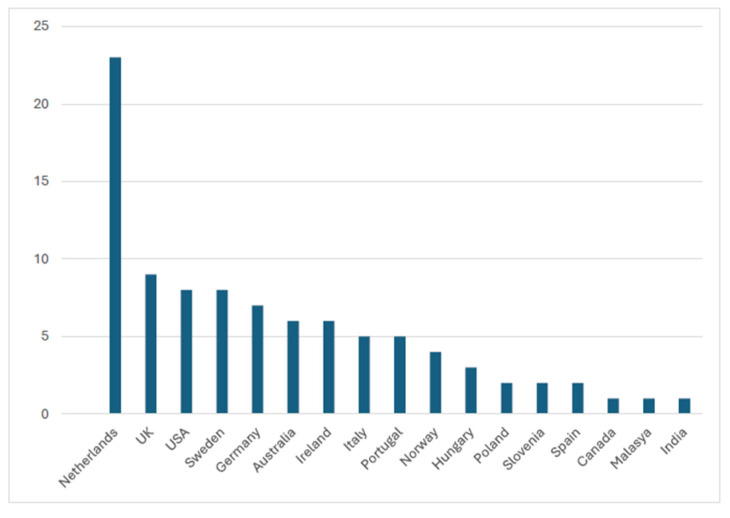
Geographical location of studies.

The instrument was applied mostly between 2017 and 2020 (both inclusive) as 62.26% of these publications had been published in the last 5 years. Most studies were carried out in the Netherlands (*n* = 23), followed by the UK (*n* = 9), USA (*n* = 8), Sweden (*n* = 8), and Germany (*n* = 7) It is important to note that in some studies, the research was carried out in several countries (Figure 2).

Only one application was found in Canada, Malaysia, and India.

### 3.1. Methodological Aspects of Analyzed Studies

Most of the manuscripts analyzed followed a cross-sectional design (74.1%) with non-probabilistic sampling (79.3%), convenience sampling being the most often used (71.7% of studies). Only 20.7% used probability sampling (simple random sampling was carried out in all studies). The sample size ranged from six participants in the Mulders-Manders et al. [38] study to 5197 participants in the Metzelthin et al. [39] study, with a mean of 471.05 participants and a standard deviation of 850.52.

Moreover, 20.75% of the studies were psychometric, i.e., they analyzed the psychometric properties of the test scores (in this case, reliability and clinical, convergent, and discriminative validity), and most of these were carried out by Hoefman et al. (*n* = 7). The remaining studies (*n* = 46) were applied in nature, focusing on caregivers and the various groups in their care [40].

### 3.2. The Characteristics of the Caregiver Samples Included in the Study

Table A2 (Appendix A.1) lists the main characteristics of the informal carers who have been included in the studies.

### 3.3. Psychometric Properties of CarerQol Instrument

#### 3.3.1. Validation Studies

Of the 54 articles included, only 12 focused on psychometric factors (see Appendix A.1, Table A3). First, the validity of the different integrated studies was analyzed based on the classification made by Hoefman et al. [31].

#### 3.3.2. Convergent Validity

Only 11 of the analyzed studies examined convergent validity (see Appendix A.1, Table A3), which assesses whether the constructs underlying CarerQol are similar to other instruments that also assess caregiver burden.

Brower et al. [21] demonstrated excellent convergent validity, revealing that greater support and satisfaction in caregiving were associated with increased happiness among caregivers. The CSI score showed a negative correlation with the CarerQol-VAS score, indicating that higher care-related quality of life was linked to lower caregiver burden. Furthermore, CSI was statistically significantly associated with all seven burden dimensions, as expected with CarerQol-7D. Similarly, SRB and PU were also significantly associated with most CarerQol 7D scores in the expected direction.

Payakachat et al. [41] also supported this validity, showing strong correlations between CarerQol-7D and CES-D (r = 0.6549) [42] and moderate correlations with SRB (r = 0.4505). The correlation between CarerQol-VAS and CES-D was (r = −0.74). In this study, the correlation coefficients were interpreted as follows, 0.00–0.19 (very weak), 0.20–0.39 (weak), 0.40–0.59 (moderate), 0.60–0.79 (strong), and 0.80–1.00 (very strong), with *p* < 0.05 indicating statistical significance [43].

Hoefmann et al. [44] confirmed these findings and found negative associations between CarerQol-VAS and caregiving burden, as well as positive associations between CarerQol-7D and caregiving support and satisfaction. Similarly, SRB, PU, and ASIS were also significantly associated with most CarerQol 7D and CarerQol-Vas scores in the expected direction (see Appendix A.1, Table A3).

Furthermore, Hoefmann et al. [45] reported similar results in caregivers of people with disabilities, demonstrating positive correlations between CarerQol-VAS and support and satisfaction, and negative correlations with the negative dimensions of CarerQol-7D. These authors also demonstrated positive correlations between the positive dimensions of CarerQol-7D and the ASIS and PU instruments, as well as positive correlations between the negative dimensions of CarerQol-7D and the SRB and CSI instruments.

Additional studies, such as those by Hoefmann et al. [31], Richters et al. [46], McLoughlin et al. [47], McCaffrey et al. [48], and Voormolen et al. [49], supported the convergent validity of CarerQol in diverse caregiver populations. In the case of McLoughlin et al. [47] and McCaffrey et al. [48], they found positive correlations between CarerQol-7D and ASCOT-Carer, as well as with EQ-5D and ICECAP-A. In Baji et al.’s [50] study, the following correlations were found: CarerQol 7-D with CarerQol-VAS (r = 0.363), CarerQol-7D with EQ-5D-5L (r = 0.453), CarerQol-7D with EQ VAS (r = 0.387), CarerQol-VAS with EQ-5D-5L (r = 0.453), and CarerQol-VAS with EQ VAS (r = 0.242).

All the studies reported good convergent validity of CarerQol, as CarerQol 7-D correlates, as expected, with CarerQol-VAS, i.e., informal caregivers reported higher levels of happiness when they felt greater satisfaction with caregiving and received support from their environment. Both parts of the instrument correlated with the other instruments, as expected, i.e., caregivers showed higher burdens or negative feelings associated with caregiving when they reported lower happiness, satisfaction, and support associated with caregiving. This correlation reinforces the validity of CarerQol as an effective tool for assessing various aspects of informal caregivers’ experiences and their implications for their emotional well-being.

#### 3.3.3. Clinical Validity

##### Age

In the study by Brower et al. [21] on caregivers of people with disabilities, age was found to have significant association with CarerQol-VAS.

##### Gender

Brower et al. [21] also reported that gender was not significantly related to CarerQol-VAS among caregivers of people with disabilities. Additionally, Voormolen et al. [49] highlighted that male gender among caregivers was associated with increased caregiver burden and reduced happiness.

##### Paid Employment

Brower et al. [21] found no significant relationship between paid employment and CarerQol-VAS among caregivers of people with disabilities.

##### Frequency of Caregiving

In the study by Brower et al. [21], CarerQol-VAS scores were negatively correlated with the frequency of caregiving, indicating that higher frequency of caregiving was associated with lower reported happiness among caregivers. Voormolen et al. [49] also found that an increase in caregiving intensity was associated with higher caregiver burden and reduced happiness. Baji et al. [50] also found that CarerQol-VAS scores were negatively correlated with frequency of care and higher caregiving burden.

##### Living with the Caregiver

Brower et al. [21] found a positive correlation between CarerQol-VAS scores and living with the caregiver.

##### Satisfaction

Hoefman et al. [31,44,45] investigated the relationship between caregiver satisfaction and CarerQol-VAS across different caregiver groups. They consistently found a positive association between caregiver satisfaction and CarerQol-VAS.

##### Relational Problems and Higher Caregiving Tasks

Hoefman et al. [31,44,45] observed a negative relationship among relational problems, higher caregiving tasks, and CarerQol-VAS.

##### Health

Voormolen et al. [49] examined caregivers of dementia patients and found that indicators of poorer caregiver and caregiving health status were associated with lower CarerQol utility scores. Baji et al. [50] also reached the same conclusions with their sample of informal caregivers.

##### Other Factors

Hoefman et al. [31,44,45] noted that institutionalization of the cared-for person, less frequent visits, and less intense caregiving tasks were related to higher levels of happiness among caregivers.

After the individual analysis of the different articles, it was concluded that the CarerQol constructs were associated with certain explanatory variables, such as hours of care, support received, gender, or work, so that it can be concluded that the instrument has good clinical validity, as the participants with similar caregiving experiences have similar scores on CarerQol and, vice versa, those with different caregiving experiences have different scores on the instrument.

#### 3.3.4. Discriminant Validity

Discriminant validity is an important characteristic of any measurement instrument, as it refers to the ability of the instrument to effectively distinguish between different groups or conditions that are supposed to be different in the variable being measured. The discriminative validity of the scores was examined in four studies [31,45,48,50]. In Hoefman et al.’s [45] study with caregivers of people with disabilities, it was observed that those who were supported and had fewer problems had higher scores on CarerQol-VAS, ASIS, and PU, while they had lower scores on SRB and CSI compared to caregivers at the opposite extreme, suggesting that CarerQol-7D can effectively differentiate between low and high burden caregivers. In another study by Hoefman et al. [31] with caregivers of terminally ill patients, it was found that groups with extremely positive evaluations experienced less burden and had higher scores on CarerQol-VAS compared to groups with extremely negative evaluations. McCaffrey et al. [48] observed moderate discriminative validity, as only higher caregiver-related scores were associated with fewer caregiving hours per week on CarerQol-7D.

In the study by Baji et al. [50], CarerQol-VAS scores were higher among caregivers who felt satisfied and received support, particularly when no issues were present. Additionally, both EQ-5D-5L [51] and EQ VAS scores were significantly higher for caregivers without mental or physical health problems.

These findings indicate that CarerQol is able to distinguish between different levels of caregiver burden and satisfaction in various situations.

After analyzing these four articles, it was seen that the instrument had good discriminative validity, as it is able to differentiate between “extreme groups” of caregivers, i.e., caregivers who had a lot of support were satisfied in caregiving and showed low scores on the negative dimensions of CarerQol [relational problems, problems associated with mental health, problems in reconciling care with daily activities, financial problems, and problems related to physical condition], while they were also happier and suffered less burden associated with caregiving.

#### 3.3.5. Feasibility of CarerQol

Feasibility is a measure that shows the percentage of scales completed by the subjects in the sample with no missing items. Brouwer et al. [21] were the first authors to conclude that CarerQol presented excellent feasibility. Based on this, 94% of the participants completed CarerQol-7D and 99% completed CarerQol-VAS (analog scale 1–10). Hoefman et al. [44] noted that the instrument measured caregiver impact in a valid and reliable way and, like Brouwer et al. [21], indicated its good feasibility, as the majority of caregivers (% NR) completed all the required questions. McCaffrey et al. [48] indicated that 99.7% of the respondents completed the questionnaire, while Mbakile-Mahlanza et al. (2019) [52] reported excellent feasibility, as 98% of the subjects completed all the items.

#### 3.3.6. Reliability Study of the Instrument

**Figure 3:**
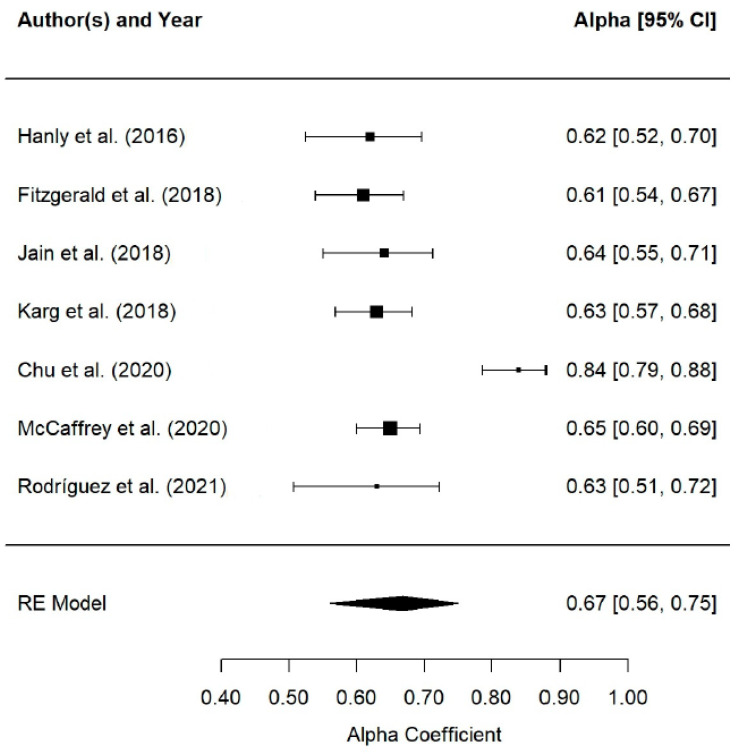
Forest Plot RG MA on Cronbach’s a coefficients [48,53,54,55,56,57,58].

**Figure 4:**
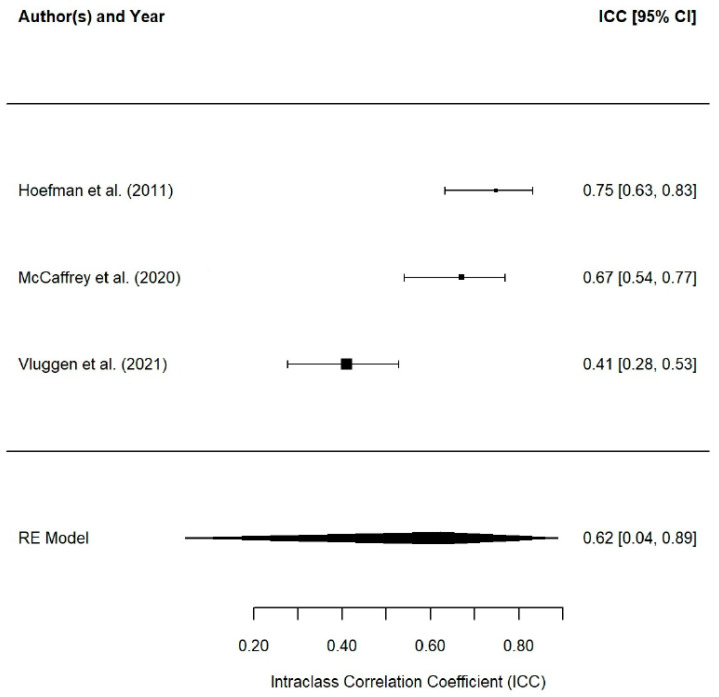
Plot RG MA on test−retest reliability coefficients [8,48,59].

The reliability of the test scores, i.e., the degree to which the instrument produces consistent and coherent results, was examined in a few studies. Of the 54 studies considered, only 9 (17%) reported reliability estimates of the test score reliability estimated on the study sample itself [48,53,54,55,56,57,58,59]. In seven studies, the reliability of the scores was estimated as their internal consistency using Cronbach’s alpha coefficients (see Figure 3) and in three studies, the temporal stability of the scores was estimated by the test–retest method using intraclass correlation coefficients (see Figure 4).

Cronbach’s α coefficient ranged from 0.61 [54] to 0.75 [53], with an average coefficient of 0.67 (95% CI [0.56, 0.75], k = 7) (see Figure 3). There was some evidence of heterogeneity among reliability coefficients (Q(6) = 31.20, *p* < 0.001, I^2^ = 88.3%).

Only three studies examined the test–retest reliability of CarerQol (see Figure 4), and all of them computed the intraclass correlation coefficients (ICCs) as a measure of the score stability. In these studies, the test–retest reliability coefficients ranged from 0.41 [59] to 0.75 [8] and presented an average coefficient of 0.62 (95% CI [0.04, 0.89], k = 3). The time interval between test–retest administrations ranged from 2 weeks [8] to 24 weeks [59], with an average of 15 weeks (SD = 12.73). Again, there was evidence of heterogeneity among the reliability coefficients (Q (2) = 17.90, *p* = 0.0001, I^2^ = 87.7%).

To examine the degree to which the results of the meta-analysis can be generalized to all the studies that used the CarerQol instrument, regardless of whether they induced or reported any reliability estimate by means of their own data, the sample characteristics (mean and standard deviation of age and percentage of men) of the studies that reported and those that did not report any estimate of reliability of scores on the CarerQol instrument were compared using separate meta-analyses. No statistically significant differences were found between these two groups in the sample characteristics.

#### 3.3.7. Valuation Studies

##### CarerQol-7D Tariffs

Several authors have also calculated tariffs that reflect social preferences for the different care states identifiable through the first part of the instrument (CarerQol-7D) [60,61]. These rates allow the caregiving states to be assessed in terms of care-related quality of life. In the calculation of the CarerQol index score, rates derived from evaluation studies conducted in the general population are used. This methodology contributes significantly to understanding the context of the valuation studies identified in this review.

The resulting scores cover the differences in the importance of the problems that carers may face. There are country-specific tariffs for different versions of the instrument, including Dutch, Australian, German, Swedish, American, English, Hungarian, Polish, and Slovenian [25], that make it easier to introduce the effect of informal care into economic evaluations.

Discrete choice experiments were carried out to calculate the tariffs for different countries, in which the participants were asked to imagine taking care of a family member with a particular condition. They were always required to keep in mind the person to whom they were providing care. The attributes and levels were taken from the first part of the instrument, which include the seven dimensions of CarerQol-7D: (1) satisfaction with caregivers, (2) relational problems, (3) problems associated with mental health, (4) problems in reconciling care with daily activities, (5) financial problems, (6) support received, and (7) problems related to physical conditions. The attribute level range assigned by the participants was “none”, “some”, and “a lot” for all levels [60,61].

Hoefman et al. [27] indicated that the most important utility dimensions of informal care situations in the Netherlands regarding the tariffs calculated in different countries were satisfaction followed by relational problems. In 2017, Hoefman et al. [60] re-measured the tariffs in five countries (Germany, Australia, Sweden, the US, and the United Kingdom), concluding that the most important dimension of usefulness was physical health. However, Germany is a bit behind these countries, as the problems associated with mental health and those related to physical conditions ranked fourth and fifth. Baji et al. [61] also examined CarerQol-7D tariffs in three Central and Eastern European countries, showing that in Hungary and Slovenia, problems related to physical conditions and those associated with mental health showed higher scores, followed by satisfaction. Satisfaction was the most important domain in Poland for those who completed the questionnaire, followed by problems associated with mental health and problems related to physical conditions. In all countries, the least important domains were problems in reconciling care with daily activities and the support received.

## 4. Discussion

The fundamental aim of this work was to provide an updated review of the main applications of CarerQol to provide researchers and practitioners with a broad overview of the instrument, to check whether its wide application in different contexts is well-reasoned, and to describe and synthesize its main psychometric properties (e.g., reliability and convergent, clinical and discriminative validity). Since its development in the Netherlands in 2006, its use in samples of informal caregivers has progressively increased, especially in the last 5 years, due to some extent to the fact that informality in care is moving out of the background of care systems [62,63]. However, this instrument is most widely used in the Netherlands (43.4%), which may be largely due to the fact that it was developed in this country.

Validity analysis of the CarerQol instrument shows that its underlying constructs are similar to other instruments measuring caregiver strain, such as CSI and the SRB scale. Studies have shown good convergent validity, indicating that informal caregivers report greater happiness and lower emotional burden when they experience job satisfaction and support [8,21,31,41,45,46,47,48,49]. In addition, CarerQol was shown to differentiate between extreme groups of caregivers, reflected in studies where those with greater support and satisfaction have higher scores on CarerQol-VAS and the positive dimensions of CarerQol-7D [45,48]. Furthermore, their constructs were linked to relevant explanatory variables such as the number of hours of caregiving and the support received, showing that caregivers with more help and fewer problems in the negative dimensions report higher satisfaction and lower burden [21,31,44,45,49]. These findings were supported by research such as that of Lutomski et al. [64].

Regarding the reliability of CarerQol, it is noteworthy that most studies (83%) did not report reliability estimates based on study-specific data, despite APA recommendations [65]. Only 17% of the studies provided such estimates, a lower percentage than reported in recent reliability generalization studies [66,67]. Reliability is not an inherent characteristic of the instrument, but of the scores obtained in a specific population [34,68]. According to the APA Task Force on Statistical Inference, authors should report reliability coefficients of the scores analyzed, even when their research does not focus on psychometric aspects [68]. Ignoring the reliability of scores may affect the estimation of effect size and statistical power in hypothesis testing [69], which highlights the importance of their reporting.

Of the studies that reported reliability, Cronbach’s alpha coefficient was the most commonly used method (77.8%), followed by test–retest reliability (33.3%, intraclass correlation coefficient). The mean Cronbach’s alpha was 0.67, below the recommended values for clinical use (>0.90) and research (>0.80), but acceptable for exploratory studies (>0.70), according to Nunnally and Bernstein [70]. Regarding test–retest reliability, there is no theoretical consensus on its interpretation, as it depends on the time interval between measurements [71,72,73]. In this study, the time interval ranged from 6 to 24 weeks, with a mean reliability of 0.62, suggesting temporal stability [72,73]. No significant differences were found between studies that reported reliability and those that did not, indicating that the generalizability of these results is valid for any research using CarerQol.

Assessment studies have generated population-based rate sets of CarerQol-7D in several countries, including Australia, Germany, Hungary, the Netherlands, Poland, Sweden, Slovenia, the UK, and the US. In all of these, all seven dimensions of CarerQol-7D were significantly associated with observed choices and attention-related utility. The areas that contributed most to the utility scores were caregiver satisfaction and mental health problems, while those that contributed least were problems balancing caregiving with daily activities and support received [50,60]. However, it is noted that countries such as Germany and the Netherlands show differences in the valuation of informal care compared to Anglo-Saxon countries and Sweden. As Baji et al. [50] point out, these differences could be due to subjective life expectations, economic differences, labor market participation, and attitudes toward informal care. In addition, family structure, infrastructure, and access to services vary between the regions of Central and Eastern Europe and the Anglo-Saxon countries [60].

### 4.1. Limitations and Future Research

#### 4.1.1. Methodological Limitations

The study has methodological limitations. The search was limited to “caregivers* AND CarerQol*”, which could have omitted some relevant articles, although it is relevant to mention that a recent study [74] used a combination of terms that only mentioned the name of the scale analyzed. Therefore, words that were synonymous with the word caregivers were not used either. This approach sought to ensure that the review remained within a clear and relevant framework, avoiding the inclusion of studies that might be less relevant or that focused on other aspects of care not directly related to CarerQol.

Regarding reliability, only nine articles provided estimates based on the study sample, which are included in the meta-analysis. The heterogeneity observed in these reliability coefficients suggests that the consistency of CarerQol may vary depending on specific characteristics of the sample, such as age, gender, type of caregiving, or cultural context. However, this study did not explicitly analyze these potential moderators, representing a significant gap in understanding the factors that influence CarerQol’s reliability.

In addition, we used Hoefman’s classification to assess validity, although the “Guidelines for Standards” lists several sources of evidence that could have been used to assess this construct. These choices may have affected the completeness of our assessment of validity.

#### 4.1.2. The Limitations of the Study

Firstly, the language limitations should be considered, including that only papers written in English or translated into Spanish might have affected the number of eligible manuscripts that applied the instrument. Despite the increasing globalization of scientific research, many of the resources and tools available for the management and analysis of scholarly articles are focused on these two languages, which also influences decision-making regarding languages of inclusion. Secondly, most studies did not estimate the reliability of the instrument scores with their sample data, so that only nine studies could be included in the meta-analysis. This might have affected the precision of the average reliability estimate, which might possibly be compromised. Also, given the scarce number of manuscripts which reported a reliability estimate, it was not possible to perform moderator analyses to search for study characteristics (such as substantive and methodological characteristics), which are statistically linked to the reliability estimates and can offer no theoretical explanations for the presence of heterogeneity [75].

#### 4.1.3. Limitations of the Instrument

The CarerQol instrument has several limitations that affect its applicability and accuracy. Firstly, it shows moderate reliability, with Cronbach’s alpha coefficients between 0.61 and 0.75, below the recommended threshold for clinical use (≥0.90), and test–retest reliability varying between 0.41 and 0.75, indicating limited temporal stability. To improve this, further studies are suggested to review and adjust items that generate variability in responses. Second, there is high heterogeneity in reliability coefficients across studies, suggesting that reliability may vary according to cultural context, type of caregiver, or population studied. Moderation analyses are recommended to identify these factors and adapt the instrument accordingly. Furthermore, only 17% of studies using CarerQol report reliability estimates based on their own data, which limits the generalizability of the results. To address this, it is suggested that future studies be encouraged to include specific reliability estimates, following APA recommendations. Finally, although CarerQol was translated into several languages, cultural differences in perceptions of care and quality of life may affect its validity in different contexts. To improve this, it is proposed to conduct cross-cultural validation studies in more countries and to adjust the items to reflect these cultural differences. These improvements could increase the accuracy and applicability of CarerQol in a variety of clinical and research settings.

#### 4.1.4. Study Strengths

This study has a number of strengths; studies involving numerous contexts, cultures, and social aspects were included to increase the generalizability of the results obtained. As Epner and Baile [76] point out, different cultural processes are involved within the same ethnic group, as there are differences in terms of age, gender, religion, among other aspects. All these factors influence the variants observed in the care of people with a disability and their caregivers, allowing us to make different comparisons between caregivers. In addition, this study is the first to include a reliability generalization study of the CarerQol instrument.

## 5. Conclusions

This study provides an innovate overview of the analysis of the CarerQol tool applied in numerous settings. The clinical convergent and discriminative validity test and the meta-analysis reveal that a large part of these studies lack certain data, which hinders analyzing their results. Only nine manuscripts reported an estimation with their own data, which is far below recent generalization reliability studies. In future research, it will be of vital relevance for other studies to report these data to find the methodological characteristics of manuscripts that are related to reliability estimates.

In conclusion, CarerQol is a valuable instrument for assessing both positive (satisfaction, support) and negative (emotional stress, financial burden, physical health problems) aspects of informal care, notable for its multidimensional approach and adaptability to diverse cultural contexts. Although its moderate reliability (average Cronbach’s alpha coefficient of 0.67) and complexity suggest caution in its use for clinical decision-making, it is especially useful in exploratory research and in specific clinical groups, such as caregivers of people with dementia, autism spectrum disorders (ASDs), chronic illness, severe disabilities, patients in palliative care, or with neurodegenerative diseases. In these contexts, CarerQol allows for the identification of specific needs and the design of personalized interventions to improve the caregiver’s well-being. However, in contexts that require quick or more specific assessments, other instruments may be preferable: the Caregiver Strain Index (CSI) is ideal for measuring emotional and physical burden in a brief manner; Self-Rated Burden (SRB) offers a quick assessment of perceived burden; the Carer Experience Scale (CES) provides a balanced view of the caregiver experience; and ASCOT-Carer focuses on social and emotional well-being.

While it can be a valuable tool for exploring caregiver quality of life, its use must be accompanied by careful consideration and interpretation of the results to ensure meaningful and accurate outcomes. It was also shown to have good clinical, convergent, and discriminant validity.

## Abbreviations

The following abbreviations are used in this manuscript:

CarerQol	Care-related Quality of Life instrument
CSI SCQ	Caregiver Strain Index
CES	Carer Experience Scale
SRB	Self-Rated Burden
ASCOT-Carer	Adult Social Care Outcomes Toolkit for Carers
PU	Process Utility
ASIS	Assessment of the informal care situation scale
ICECAP-A	ICEpop CAPability measure for Adults

## Appendix A

Table A1

Table A1 lists the main results and characteristics of the 53 studies analyzed.

Table A2

Table A2 contains the main characteristics of the caregiver samples included in the study.

Table A3

Table A3 exclusively contains articles measuring the psychometric properties of the instrument.

Table A4

Weighted ANOVAs results performed on Sample Characteristics of the Studies That Reported and Induced Reliability estimate of Test Score.

Table A5

A checklist for the corroboration of the meta-analytical report according to REGEMA. The REGEMA checklist is a tool composed of 30 items (1 item for the Title, 1 for the Abstract, 2 for the Introduction, 14 for the Methods, 6 for the Results, 4 for the Discussion, 1 for Funding, and 1 for Protocol). REGEMA can be applied both by researchers that intend to write an RG meta-analysis and to make a critical reading of RG meta-analyses, as well as for editors and reviewers as a tool to assess the adequacy of an RG meta-analysis.

Table A6

The PRISMA statement comprises a 27-item checklist addressing the introduction, methods, results, and discussion sections of a systematic review report.

### Appendix A.1

**Table A1:** The main findings of the studies included in the review (*N* = 53).

Author and Year	Location	Sample	Research Design and Sampling	Cared Group	Kind of Caregiver	Average Age (of)	Gender	Ethnicity	Level of Education	Type of Study	Results	Limitations
[21]	Netherlands	175 (regional centers)	Cross-sectional design; sampling NP (for convenience)	NR	Informal	60.8 (13.1)	M:25%; W:75%	NR	Low: 45%/Medium: 34%/High: 21%	Psychometric	eExcellent viability of the instrument. Excellent convergent validity between the relationships of the two parts of the CarerQol instrument, CSI, SRB, and PU.	The reliability of the instrument was been measured. Relatively short study.
[8]	Netherlands	230 (regional carer support centers)	Cross-sectional design; P sampling (simple random)	Sick and/or disabled people	Informal	58.74 (12.74)	M:25.7%, W:74.3%	NR	Low:13.1%/ Medium: 61.6%/ High:25.3%	Psychometric	After measuring clinical and convergent validity (associations with SRB and PU), the CarerQol instrument details the effect of caregiving.	Non-representative sample of the Dutch informal carers population. Lack of response.
[44]	Netherlands	108 (people using nursing home care)	Longitudinal design; NP sampling (for convenience)	Elderly people	Informal	59.1 (11)	M:28.6%; W:71.4%	NR	Low: 16.5%/Medium: 56.7%/High:29.5%	Psychometric	The CarerQol instrument assesses the effect of care in a feasible, valid, and reliable way. Shows good convergent (SRB and ASIS) and clinical validity.	Small sample size. Lack of responses.
[41]	US	65 (Arkansas Reproductive Health Monitoring System)	Cross-sectional prospective design; NP sampling (for convenience)	Children with cranioencephalic malformations	Informal	31.9 (5.3)	M:1.6%; W: 98.4%	Caucasian: 89.2%/Afro-American:6.2% /Other:4.6%	Low:24.6%/ High:76.4%	Psychometric	Acceptable construct validity; moderate associations of CarerQol-7D and CES-D, and high negative correlations between CarerQol-VAS and CES-D.	Small sample size.
[77]	Netherlands	80 (home ventilation centers)	Cross-sectional design; NP sampling (for convenience)	Adults with Duchenne muscular dystrophy	Informal	57 (6.8)	M: 31%; W:69%	NR	Low:68% High: 32%	Applied	Substantial burden, which was associated with the support received, the tracheotomy, coping, and anxiety. However, care provision is valued as rewarding.	The cross-sectional design only allows associations to be detected. Sample selection bias. Findings not applicable to other populations.
[78]	Sweden	118 (psychiatric outpatient centers)	Cross-sectional design (descriptive and methodological); NP sampling (for convenience)	Patients with psychosis	Informal	58 (15)	M:33% W:67%	NR	NR	Applied	Caregivers perceived a greater burden and underestimated the time spent on caregiving. This situation caused them psychological distress.	A comparative group was not used.
[45]	Netherlands	1244 (selected from general population)	Cross-sectional design; P sampling (simple random)	People with disabilities	Informal	NR	M:41.7%; W:58.3%	NR	Low:14.6%/ Medium:55.9%/ High:29.6%	Psychometric	This study confirmed the findings of previous studies regarding clinical validity and convergent and discriminative validity, expanding the sample of caregivers.	Selection bias. Validation is an ongoing process, and it is desirable to test psychometric properties among caregivers in other settings.
[79]	Netherlands	67 (by center)	Cross-sectional design; P sampling (simple random)	Patients suffering from Pompe	Informal	NR	M:40%; W:60%	NR	NR	Applied	Greater burden associated with service hours; higher with patients with lower QoL. Caregivers reported mental health and ADL problems. However, they reported receiving satisfaction from care delivery.	Reduced number of patients. Selection bias.
[26]	USA	310 (recruited from different associations)	Cross-sectional study; NP sampling (for convenience)	Patients suffering from hemophilia	Informal	NR	M:10.7% W:89.4%	NR	Low:11.0%/ Medium:26.5%/ High:62.6%	Applied	Caregivers of children with inhibitors had a higher burden than those without inhibitors. Those caregivers of patients with inhibitors noticed a lower QoL.	Bias in study results.
[80]	USA	304 (recruited from different associations)	Single assessment cross-sectional study; NP sampling (for convenience)	Patients suffering from hemophilia	Informal	NR	G 1: M:20%/W:80% G2: M:9.9%/W:90.1%	NR	Group 1: Medium:6.7%/High:93.3%//Group 2: Low: 0.7%/Medium: 10.6%/High: 88.7%	Applied	Caregivers reported limited burden. However, caregivers reported satisfaction with care tasks.	Responses not representative. Bias in results.
[27]	Netherlands	992 (adult population of the Netherlands)	Cross-sectional design; sampling P (simple random)	NR	Informal	49.2 (16)	M:39.9% W:60.1%	NR	Low: 19.1%/Medium: 52.7%/High: 28.2%	Applied	The most important usefulness dimensions of care situations for this country were satisfaction followed by relational problems. Physical health is more substantial if coupled with mental health problems.	The group in this study is not considered representative of the population of the Netherlands, as women were overrepresented.
[80]	US	224 (two autism treatment network sites)	Cross-sectional design; NP sampling (for convenience)	Children with ASD	Informal	39.4 (8.3)	M:11% W:89%	NR	Low: 12% High:88%	Applied	Carers’ burdens were lower when they received support, were satisfied with caregiving, and felt well. In contrast, they were higher when there were problems related to mental health, problems related to physical health, financial problems, and problems in balancing caregiving with daily activities.	Overrepresented patients. Bias in results.
[81]	Netherlands	159 (by patients)	Longitudinal design; sampling P (simple random)	Elderly with frailty	Informal	EG: 60.7 (12.2)/CG:65.6 (11.2)	M:27% W:73%	NR	Experimental: Low: 65.4%/High: 34.6% Control: Low:(66.2%)/High (33.8%)	Applied	CarerQol showed that there was a reduction in subjective burden, and that carers had fewer problems as a result of the intervention.	Low proportion of variance explained by the intervention. The control variables had a low contribution. Lost to follow-up.
[82]	Netherlands	223 (Netherlands’ register of dementia carers)	Transverse design; NP sampling (for convenience)	Patients suffering from dementia	Informal	66.4 (13.4)	M:34.5% W:65.5%	NR	Low: 12.6%/Medium: 58.7%/High:28.7%	Applied	Caregivers experience certain problems in the relationship with the patient, the combination of ADL, and with their own mental health. However, the majority experienced satisfaction in the care and support of the immediate circle.	Small and selective sample.
[83]	USA	224 (centers for children with ASD and their caregivers)	Cross-sectional design, with a triangulation mixed-methods approach; NP sampling (for convenience)	Children with autism spectrum disorder (ASD)	Informal	39.4 (8.3)	M:10.5% W:89.5%	Caucasian:72.8%/African American:9.4%/Hispanic:10.8%	Low:8.5% High:91.5%	Applied	The health of the caregivers was lower than the normative population. The caregivers of these children suffered from social anxiety and stress.	There is no control group. No information is provided about the income of the family.
[84]	Spain	202 (registry of caregivers)	Cross-sectional design; sampling P (simple random)	NR	Informal	47.8 (12.8)	M:31.7% W:68.3%	NR	Low: 45.1%/Medium:28.2%/High:26.8%	Applied	A monetary valuation was carried out between caregivers and non-caregivers, showing that it is possible to receive a monetary value for informal care from the non-carers’ preferences.	Relatively small sample size. No representation of caregivers.
[31]	South Australia	97 (carers associated with Southern Adelaide Palliative Services)	Cross-sectional design; NP sampling (for convenience)	Patients at the end of life	Informal	62.3 (11.9)	M:29% W:71%	Australian/European: 98%/Asian:1%	NR	Psychometric CarerQol	Shows good convergent validity (PU and CSI), clinical validity, and discriminative validity.	The sample size was small. Caregivers were predominantly older women.
[85]	USA	224 (treatment centers)	Cross-sectional, prospective design study; NP sampling (for convenience)	Children with autism spectrum disorder (ASD)	Informal	39.4 (8.3)	M:10.5% W:89.5%	Caucasian: 72.8%/African American:9.4%/Hispanic: 10.8%/Asian:3.3%/Other: 3.3%	NR	Applied	Negative effects on QoL and well-being associated with sleep problems in children. Caregivers who slept ≤ 5 h showed scores that suggested the presence of more depressive symptoms and worse QoL.	Cross-sectional design to quantify QALYs. Direct measures were not used in certain processes in the study.
[55]	Ireland	180 (caregivers of people enrolled in the Irish National Cancer Registry)	Cross-sectional design study of a descriptive and correlational nature; NP sampling (for convenience)	Patients with head and neck cancer	Informal	57.3 (12.5)	M:24% W:76%	NR	NR	Applied	Caregivers experienced satisfaction with their care tasks and high levels of happiness. Almost half reported mental and physical health problems.	Cross-sectional nature indicates that no causality claims can be made. CarerQol-VAS was not specific to cancer carers.
[86]	Netherlands	356 (test phase); 158 (post-test phase) (through the organizations of the Elderly Care Network)	Longitudinal design; NP sampling (convenience)	Older adults	Informal	63.2 (11.4)	M:32% W:68%	NR	NR	Applied	Carers reported problems related to physical and mental conditions as well as daily activities. The burden was higher when the cared-for person’s health was worse.	Limitation when it comes to generalizing the results.
[87]	UK	NR	Longitudinal design/probability (random) sampling	Duchenne muscular dystrophy	Informal	NR	NR	NR	NR	Applied	This study lacks necessary information on valid therapeutic protocols for people with DMD and their caregivers.	NR.
[60]	Australia, Germany, Sweden, UK, and USA	Australia: 551/Germany: 562/Sweden: 548/UK: 552/USA 550	Cross-sectional design; NP sampling (for convenience)	Care	scenarios Informal	Australia:45.5 (16.4)/Germany:46.7 (16.3)/Sweden:47.3 (17.5)/:UK:46.6 (16.7)/USA: USA:45.8(16.9) years	M:Australia:48.8/Germany:48.4/Sweden:49.1/UK:48.4/USA:48.6% W:Australia:51.2/Germany:51.6/Sweden:50.9/UK:51.6/USA:51.5%	NR	Low: Australia:1.1/Germany:21.2/Sweden:17.9/UK:20.7/USA:13.3// Medium: Australia:67.7/Germany:55.7/Sweden:49.6/UK:23/USA:28.2//Raised:Australia:31.2/Germany:23.1/Sweden:32.5/UK:56.3/USA:58.6	Applied	The most important utility dimension for caregiving situations was physical condition, but the least important dimensions were support and difficulty in combining care with daily activities.	Heterogeneity in preferences for care situations. The study sample could be somewhat selective.
[88]	Germany, Italy, Ireland, Norway, Netherlands Sweden, Portugal, and the UK	453 (campaigns, case managers, general practices, memory clinics, and community teams)	Cross-sectional prospective cohort design; NP sampling (for convenience)	Patients suffering from moderate or severe dementia	Informal	66.4 (13.3)	M:33.4% W:66.6%	NR	NR	Applied	Married women had a higher burden compared to other family members. Adaptation to change was associated with better health.	Determination of causality is limited. Cultural backgrounds in different countries may influence caregiver behaviors.
[39]	Netherlands	5197 (NR)	Cross-sectional design; NP sampling (for convenience)	Older adults	Informal	80.7 (7.25)	M:38% W:62%	94% native	Low:44%/ Medium:51%/ High:5%	Applied	Caregivers produced a high burden and reduced QoL. This situation was accentuated in female carers.	Problems with generalizability of results. Relatively high non-response on some items.
[89]	Netherlands	660 (NR)	Longitudinal design; NP sampling (for convenience)	Elderly people with frailty	Informal	65 (12.6)	M:32% W:68%	NR	NR	Applied	A worse symptomatology of the patient was related to a worse QoL of the caregiver and with the presence of more problems related to mental and physical conditions, and with combining care with daily activities.	Abandonment of certain subjects in the sample. Selection bias.
[46]	Netherlands	1 = 198 /2 = 166 (1. Regional Assessment Agency; 2. Community Caregivers)	Study 1: longitudinal; Study 2: cross-sectional; NP sampling (for convenience)	Patients with dementia	Informal	: 66.6 (12.9)/Study 2: 49.5 (14.4)	M: E1:1:33%/E2:45% W: E1:67%/E2: 55%	NR	NR	Psychometric	Good construct validity between the associations of CarerQol with SRB and CSI.	Divergence between the two study populations.
[9]	Sweden	97 (newspapers, social networks, and organizations with interests in caregivers)	Longitudinal design; NP sampling (for convenience)	Patients with mental illness	Informal	NR	M:11% W:89%	NR	Medium:13%/High:83%/Other:4%	Applied	Caregiver burden, in most aspects, did not show significant improvements after the intervention, except in the relational dimension.	Some participant dropouts. It is difficult to know whether any confounding factors may have influenced the results.
[90]	Sweden	151 (NR)	Longitudinal design; NP sampling (for convenience)	People suffering from mental illness	Informal	54 (NR)	M:EG:10%/CG:14% W:EG:90%/CG:86%	NR	EG: Medium:14% /High:82% /Other:4%//CG: Medium:22% /High:73% /Other:5%	Applied	The comparisons between the groups before and after the intervention showed improvements in favor of the experimental group in terms of relational problems, problems related to mental health status, and daily activities.	Dropout rate. It is difficult to know whether any confounding factors may have influenced the results.
[91]	Netherlands	350 (via care recipients)	Cross-sectional design; sampling NP (snowball)	NR	Informal	63 (13.3)	M:33.8% W:66.2%	NR	NR	Applied	Many carers stated that they would be less happy if the care of the dependent person was performed by someone else. QoL depended on variables such as health, disease deterioration, or happiness.	Overestimation of caregiver quality of life in informal caregivers.
[92]	Netherlands	123 (through care recipients)	Cross-sectional study; P sampling (simple random)	Elderly patients with hip fracture	Informal	64.6 (12.2)	M:44.7% W:55.3%	NR	Low:30.1%/Medium: 45.5%/High:24.4%	Applied	Those patients who showed cognitive impairment had a lower QoL. Relational problems were experienced by caregivers. Women have a higher burden.	Non-response bias. The cross-sectional nature indicates that causality claims cannot be made.
[54]	Ireland	326 (caregivers enrolled in the Irish Comparative Outcomes Study)	Cross-sectional design; NP sampling (for convenience)	Children suffering from cystic fibrosis	Informal	M: = 35.5 (4.8), H = 38.0 (5.4)	M:42.02% W:57.98%	NR	High: Women (68%) and Men (53.9%)//Medium: Women (32%) and Men (46.1%)	Applied	A large proportion of caregivers reported mental health problems. People who are severely depressed adhere less well to treatment. Burden on caregivers increases as children age.	The instrument is not valid for caregivers of people with CF, and it is the first time it has been used in this population. Limitation of sample.
[93]	Germany, Italy, Ireland, Norway, Portugal, the Netherlands, Sweden, and the UK	451 (NR)	Cross-sectional design; P sampling (simple random)	Patients with dementia	Informal	66 (13)	M:33% W:67%	NR	NR	Applied	Non-medical costs (i.e., social services and informal care) were a relatively great proportion of costs/QoL of caregivers was reduced.	Representativeness limited by relatively small sample sizes from each country.
[56]	Canada	181 (different centers in Canada)	Cross-sectional prospective cohort design; sampling NR (for convenience)	Children with drug-resistant epilepsy	Informal	NR	M:16% W:84%	NR	High: 78.5%/ Low: 21.5%	Applied	QoL of caregivers was higher when QoL of patients was favorable. In contrast, a lower QoL was linked to the occurrence of depressive and anxious symptoms.	NR.
[57]	Germany	386 (Bavarian Compulsory Health Insurance Fund Medical Service)	Cross-sectional design; NP sampling (by trial)	Patients with dementia	Informal	61.3 (12.2)	M:24% W:76%	NR	NR	Applied	Carers of people with dementia showed a high subjective burden and high levels of depression.	Certain care-related variables that might be of interest were not measured. The cross-sectional nature indicates that no claims of causality can be made.
[94]	Netherlands, Norway, Germany, the UK, Sweden, Ireland, Italy, and Portugal	451 (recruited from memory clinics and community mental health teams)	Cross-sectional prospective cohort design; NP sampling (convenience)	Patients with dementia	Informal	66.4 (13.3)	M:33% W:67%	NR	NR	Applied	The greater the needs, the worse the quality of life.	Selection bias.
[38]	The Netherlands	6 (via Radboud University Medical Center and Erasmus Medical Center)	Observational study, cross-sectional design; NP sampling (convenience)	Patients suffering from cryopyrin-associated periodic syndrome	Informal	NR	NR	NR	NR	Applied	Informal caregivers showed a higher subjective burden than the general Dutch population. They also indicated mental health-related problems due to the care provided.	Relatively small sample.
[95]	Hungary, Poland, and Slovenia	395 (NR)	Cross-sectional design; NP sampling (by quotas)	Hypothetical care situations	Informal	Hungary: 56.1 (NR)/Poland: 45.6 (NR), Slovenia:48 (NR)	M: Hungary:41.6%/Poland:50%/Slovenia:49% W: Hungary: 58.4%/Poland:50%/Slovenia:51%	NR	:HU:18.8%/PO:7.3%/ES:11.5%/Medium:HU:36.9%/PO:60, 7%/ES:61.5%/ High:HU:44.3%/PO:32.0%/ES:27.1%	Applied	Most caregivers were satisfied with the care. In both Hungary and Slovenia, problems in combining care with ADL were the most prominent. Women were more likely to be caregivers in Hungary.	Over-representation of certain population groups.
[52]	Australia	40 (general and psychogeriatric nursing homes)	Longitudinal design; NP sampling (for convenience)	Patients suffering from dementia	Informal	Group A: 67.7 (12.6)/Group B: 59.6 (6.8)	M:15% W:85%	NR	Group A: Low (10%) Medium: (12.5%) High: (27.5%) Group B: Low(22.5%) Medium: (12.5%) High: (15%)	Applied	The effect on the caregivers after the intervention was limited and there were no major changes among the conditions for the quality of the relationship of caregiver–person cared-for and caregivers’ sense of mastery and reported QoL.	Small sample size. Differences in the characteristics of the caregivers in both groups.
[96]	UK and USA	56 (NR)	Observational study with a cross-sectional design; P-sampling (random)	Patients with degenerative cervical myelopathy	Informal	NR	M:40% W:60%	White:98%/African American:2%	Low:11%/ Medium:52%/ Superior: 37%	Applied	CarerQol only measured caregivers’ happiness, which reported high values.	Relatively small sample.
[61]	Hungary, Poland, and Slovenia	*n* = 1000 HU/*n* = 1000 PO/*n* = 1000 ES (NR)	Cross-sectional design; NP sampling (by quotas)	Care	Informal	53.2 ± (15.1) Hungary, 45.1 ± (15.7) Poland 46.4 ± (16.0) Slovenia	M:48.8% HU/47, 5%PO/47.6% ES W: 51.2% HU/52.5%PO/52.4% ES	NR	Hungary: Low:23.1%/Medium:37.4%/High:39.5%//Poland: Low:11.1%/Medium: 66.7%/High:22.2%//Slovenia: Low:17.6%/Medium:55.8%/High:26.6%	Applied	In Hungary and Slovenia, physical health and mental health problems contributed the most to the utility score, followed by satisfaction. In Poland, satisfaction was the most important domain.	Sample selection bias and standard errors.
[97]	East India	324 (thalassemia outpatient department (OPD))	Observational study with cross-sectional design; NP (convenience) sampling	Older children with beta-thalassemia	Informal	31.8 (6.3)	M:24.1% W:75.9%	NR	Low: 66.4%/ Medium:19.8%/ High: 13.8%	Applied	Thalassaemia generates stress and strain that affects the quality of life of support persons. The caregivers of these people had a significantly high burden due to caregiving.	The cross-sectional design did not allow causal associations to be established. Much of the data were self-reported by caregivers.
[98]	Portugal	36 (caregivers of Body & Brain project participants)	Longitudinal design; NP (convenience) sampling	Patients with dementia	Informal	64.94 (13.54)	M:58.29% W:41.71%	NR	Medium: 27.8%/High:72.2%	Applied	Caregiver burden increased significantly during home confinement. Self-assessed well-being of caregivers decreased. Decreased perceived satisfaction and support.	Small sample size. Difficulty in generalizing the results.
[53]	Malaysia	110 (caregivers attending therapy at the University of Malaysia Speech and Audiology Clinic)	Cross-sectional design; NP sampling (for convenience)	Children with autism spectrum disorder (ASD)	Informal	NR	M:27.3% W:72.2%	Malays (93.6%)/Chinese/(5.5%)/Indians (0.9%)	Low:0.9%/ Medium:18.2%/High:77.3%/ Others:3.6%	Applied	The main drawback is related to relational problems with the child and mental health problems. Happiness levels reported by caregivers were moderate.	NR.
[99]	Australia	351 (through Carers Victoria non-profit organization)	Cross-cutting design; sampling NP (by trial)	NR	Informal	53	M:20% W:80%	NR	Low: 18%/ Medium: 8%/ High:73%/ Other:1%	Psychometric	Factor analysis indicates that the measures of the different instruments assess different aspects and provide unique information (CarerQol, ASCOT-Carer, CES).	Inconsistent responses.
[100]	Australia	43 (via Myeloma Australia)	Cross-sectional design; sampling NP (for convenience)	Patients suffering from multiple myolemma	Informal	NR	M:30.24% W:69.76%	NR	NR	Applied	The subjective burden experienced by caregivers of patients with multiple myolemma was relatively high. The well-being experienced by the caregivers was medium.	Sampling problems.
[101]	UK, Italy, and Germany	585 (NR)	Cross-sectional design; NP sampling (for convenience)	Patients with atrial fibrillation	Informal	46.8 (15.6)	M:42.2% W:57.6%	NR	Low: 4.3%/Medium: 46.2%/High:49.6%	Applied	Most carers expressed satisfaction with care. Many of them had problems affecting their mental and physical health and also reported problems in ADL.	Sample made up of self-reported caregivers. Selection bias.
[48]	Australia	500 (carers registered with Carers Victoria)	Cross-sectional study; sampling P (random)	NR	Informal	52 (14)	M:21% W:79%	80% Australians	Low: 18%/ Medium:10%/ High:71%/ Other:1%	Psychometric	Good discriminative and convergent validity, analyzed through the associations of CarerQol with ASCOT and CES-D.	NR.
[47]	UK	576 (via NatCen Social Research)	Longitudinal design; NP sampling (by trial)	Dementia patients	Informal	62 (11)	M:35% W:65%	NR	NR	Psychometric	Good convergent validity, analyzed between the correlations of CarerQol with ASCOT, EQ-5D-5L, and ICECAP.	Loss of subjects throughout the study.
[102]	Netherlands	81 (caregivers more involved in in the care of children with autism spectrum disorder)	Multicentre prospective cross-sectional design study; NP sampling (for convenience)	Children with autism spectrum disorder (ASD)	Informal	NR	NR	NR	NR	Applied	Caregivers reported problems with the cared-for child and complexity in combining care with their daily duties. The QoL of caregivers is lower. However, they reported satisfaction and support with care.	Relatively small sample. The subjects in this study are not characteristic of the total group of children with ASD and their support persons.
[50]	Hungary	149 (NR)	Cross-sectional study; sampling P (random)	NR	Informal	56.1 (14.2)	M:41.6 W:58.4	NR	Low: 44.3%/Medium: 36.9%/High: 44.3%	Psychometric	Good convergent, clinical, and discriminant validity of the Hungarian version of CarerQol and supports the cross-cultural. validity of the instrument.	Sampling problems.
[103]	Netherlands	42 (through organizations working with people with dementia and their carers)	Longitudinal design; NP sampling (for convenience)	Patients with mild dementia	Informal	62.8 (13)	M:26.2% W:73.8%	NR	Low: 50%/Medium: 30%/High: 20%	Applied	Caregivers did not perceive much distress and reported relatively high QoL.	Bias in the study sample.
[58]	Spain	110 (NR)	Cross-sectional design; NP sampling (for convenience)	Children with neuromuscular diseases	Informal	44.7 (7.2)	M:17.2% W:82.8%	NR	Low:13.7%/Medium: 24.6%/High:61.7%	Applied	Children with a higher level of dependency generated higher costs. The most affected carers are those who are unemployed, those with low sources of income, and those whose children had a high level of dependency. Women had the highest score in somatic symptoms.	Non-homogeneous groups. Cause and effect cannot be inferred.
[59]	Netherlands	172 (caregivers of patients attending geriatric rehabilitation units)	Longitudinal design; P sampling (simple random)	Patients who have suffered stroke	Informal	Intervention group: 61.0 (13.5)/Usual care group: 60.5 (13.5)	M: Intervention group: 35.4%/Usual care group: 29.3%//W: Intervention group: 64.6%/Usual care group: 70.7%	NR	NR	Applied	The integrated program resulted in a decrease in caregiver burden. Stroke education helps informal caregivers fulfill their support role.	Sampling bias.
[49]	Germany, Italy, Norway, Sweden, Portugal, the Netherlands, the UK, and Ireland	433 (NR)	Prospective longitudinal design; NP sampling (for convenience)	Dementia patients	Informal	66.2 (13.4)	M:34% W:66%	NR	Low (56.3%)/High (43.7%)	Psychometric	The results support that CarerQol shows good clinical and convergent validity (associations of CarerQol with ICECAP and EQ-5D).	Convenience sampling may have underrepresented burdened caregivers who were unable to participate in this study.

Note: NR: not reported; NP: non-probabilistic: P: probabilistic; CG: control group; EG: experimental group; M: men; W: women; QoL: quality of life; ADL: activities of daily living. QALYs = Quality adjusted life years; CSI: The Caregiver Strain Index; SRB: the Self-Rated Burden scale; PU: Process Utility Measure; ASIS: Informal Care Situation Assessment Scale; CES-D: Center for Epidemiological Studies Depression Scale; ASCOT-Carer: Adult Social Care Outcomes Toolkit; EQ-5D-5L: the new five-level version of EQ-5D (EQ-5D-5L); ICECAP-A: Investigating Choice Experiments Capability Measure for Adults; ASD: autism spectrum disorder; DMD: Duchenne muscular dystrophy; CF: cystic fibrosis.

**Table A2:** Main characteristics of caregivers.

Type of caregiver	In all studies (*n* = 54), caregivers were informal; formal caregivers were not included.
Caregivers’age	Mean: 58.7 years (SD = 11.92). Range: 31.8 years [97] (caregivers of children with beta-thalassemia) to 80.7 years [39] (caregivers of adults with disabilities). Younger caregivers were mostly parents, while older caregivers were often spouses.
Caregivers’gender	In 92.5% of the studies, female caregivers were the majority. A total of 5.66% did not report gender [104]. Only 1.9% of the studies had more male caregivers (58.3% caring for people with dementia) [98]. The most significant gender gap was found in Payakachat et al., with only 1.8% male caregivers [41].
Educational	Education was reported in 62.26% of the studies. A total of 28.25% had a “low” education level (primary school), 39.68% had a “middle” level (secondary school), and 31.94% had higher education. A total of 0.13% were classified as “others”.
Caregivers’ethnicity	Only 15.1% of the studies (*n* = 8) reported ethnicity. Most (*n* = 7) had a higher percentage of White/Caucasian individuals. In Chu et al., 93.2% were Malaysian, 5.5% Chinese, and 0.9% Indian [53].

**Table A3:** Psychometric properties of the instrument.

Author	Sample (n) and Gender Distribution	Psychometric Properties	Measurement of Convergent	Validity Background Variable Clinicals	Validity Discriminative Validity
[21]	*n* = 175 caregivers (Mixed)	Feasibility, clinical and convergent validity	Spearman’s correlation with CSI relational problems and CSI (*r* = 0.49), mental health problems and CSI (*r* = 0.42), problems with activities daily problems and CSI (*r* = 0.50), financial problems and CSI (*r* = 0.28), and physical problems and CSI (*r* = 0.40). Satisfaction and CSI (*r* = −0.20); 2. support and CSI (*r* = −0.21)	Frequency of care and coexistence.	NR
[41]	*n* = 65 caregivers (Mixed)	Convergent validity	Spearman’s rho with CES-D CarerQol-7D with CES-D (*r* = 0.65) CarerQol-VAS with CES-D (*r* = −0.74)	NR.	NR
[44]	*n* = 230 caregivers (Mixed)	Convergent validity, and clinical validity	Spearman’s correlation with SRB and PU CarerQol-7D with CarerQol-VAS: relational problems (*r* = −0.34); problems associated with mental health (*r* = −0.56); problems in reconciling care with activities of daily living (r = −0.44); economic problems (r = −0.19); problems related to physical condition (r = −0.44). CarerQol-VAS with SRB (r = −0.45) SRB with satisfaction (r = −0.27); and 2. support (r = −0.03). PU with satisfaction (r = 0.37) and 2. support (r = 0.15)	Satisfaction, burden, tie, and intensity.	NR
[8]	*n* = 108 caregivers (Mixed)	Feasibility, convergent validity, and clinical validity	Spearman’s correlation with SRB y ASIS CarerQol-VAS and satisfaction (r = 0.15) and support (r = 0.05) SRB and support (r = −0.06) ASIS and satisfaction (r = 0.27) and support (r = 0.13)	Tie, age, years caring, intensity, remuneration, and education.	NR
[45]	*n* = 1244 caregivers (Mixed)	Convergent validity, clinical validity, and discriminative validity	Spearman’s correlation with SRB, ASIS, PU y CSI CarerQol-VAS, and satisfaction (r = 0.24) and support (r = 0.14) ASIS and satisfaction (r = 0.24) and support (r = 0.13) and with CarerQol-VAS (r = 0.31) PU with satisfaction (r = 0.31), 2. support (r = 0.09) and CarerQol-VAS (r = 0.52) SRB with relational problems (r = 0.35), problems associated with mental health (r = 0.39), problems in reconciling care with activities of daily living (r = 0.47), economic problems (r = 0.30) and problems related to physical condition (r = 0.42) SRB with CarerQol-VAS (r = −0.33) CSI with relational problems (r = 0.38) problems associated with mental health, (r = 0.47), problems in reconciling care with activities of daily living (r = 0.52), economic problems (r = 0.42) and problems related to physical condition (r = 0.48); the relationship with CarerQol-VAS was negative (r = −0.40).	Gender, job, and health.	YES
[31]	*n* = 97 caregivers (Mixed)	Convergent validity, clinical validity, and discriminative validity	Spearman’s correlation with PU, CSI CarerQol 7-D with CarerQol-VAS (*r* = 0.60) CarerQol-7D and PU (*r* = 0.62) CarerQol-7D and CSI(+) (*r* = 0.55) CarerQol-7D and CSI(−) (*r* = −0.67)	Years of care, caregiver and patient health.	YES
[46]	*n* = 198 y *n* = 166 caregivers (Mixed)	Convergent validity	Spearman’s correlation with SRB y CSI (NR)	NR.	NR
[99]	*n* = 351 caregivers (Mixed)	NR	NR	NR.	NR
[48]	*n* = 500 caregivers (Mixed)	Convergent and discriminative validity	Spearman’s correlation with ASCOT y CES-D CarerQol-7D and ASCOT-Carer (*r* = 0.54) CarerQol-7D and CES-D (*r* = 0.45)	NR.	YES
[47]	*n* = 576 caregivers (Mixed)	Convergent validity	Spearman’s correlation with ASCOT, EQ-5D-5L y ICECAP CarerQol with ASCOT-Carer (*r* = 0.71), with EQ-5D-5L (*r* = 0.0.51) and with ICECAP-A (*r* = 0.69)	NR.	NR
[50]	*n* = 149 caregivers (Mixed)	Convergent validity, and clinical validity	Spearman’s correlation with EQ-5D-5L and EQ VAS CarerQol 7-D with CarerQol-VAS (*r* = 0.363) CarerQol-7D with EQ-5D-5L (*r* = 0.453) CarerQol-7D with EQ VAS (*r* = 0.387) CarerQol-VAS with EQ-5D-5L (*r* = 0.453) CarerQol-VAS with EQ VAS (*r* = 0.242)	Care situation, caregiver and patient health, and years of care.	YES
[49]	*n* = 433 caregivers (Mixed)	Convergent validity, and clinical validity	Spearman’s correlation with ICECAP y EQ-5D CarerQol-VAS and CarerQol-7D: 1 satisfaction (*r* = 0.34) y 2. support (*r* = 0.15) EQ-5D and satisfaction (*r* = −0.09) and support (*r* = −0.13)	Gender, intensity, and health.	NR

**Table A4:** Weighted ANOVA results performed on sample characteristics of studies that reported and induced reliability estimate of test score.

				95% CI		
Outcome	*k*	N	Average	LL	LU	ANOVA Results
Mean age (years)						*F*(1, 37) = 0.54, *p* = 0.468 *R*^2^ = 0.0 *Q*_W_(37) = 51855.57.3, *p* < 0.0001
Reported	7	1782	53.08	44.45	61.70	
Not reported	32	19,453	56.52	52.49	60.56	
Variance age (years)						*F*(1, 37) = 1.03, *p* = 0.317 *R*^2^ = 0.0 *Q*_W_(37) = 3756.19, *p* < 0.0001
Reported	7	1782	125.09	74.37	175.82	
Not reported	32	19,453	153.18	129.22	177.14	
Male (%)						
Reported	9		25.39	18.13	34.33	*F*(1, 48) = 0.79, *p* = 0.378 *R*^2^ = 0.37 *Q*_W_(48) = 844.47, *p* < 0.0001
Not reported	41		29.57	25.51	33.99	

Note: *k* = number of studies. N = sample size. 95% CI = 95% confidence Interval for average. LL = lower 95% confidence limit for average. LU = upper 95% confidence limits for average. *F* = statistic of Knapp–Hartung for assessing moderator variable significance. *Q*_W_ = statistic for assessing model misspecification. *R*^2^ = proportion of variance accounted for by moderator.

**Table A5:** A checklist for the corroboration of the meta-analytical report according to REGEMA.

TITLE	Yes	No	Page	NR
1. Title	x		1	
**ABSTRACT**	Yes	No	Page	NR
2. Abstract	x		2	
**INTRODUCTION**	Yes	No	Page	NR
3. Background	x		3	
4. Objectives	x		7	
**METHOD**	Yes	No	Page	NR
5. Selection criteria	x		7	
6. Search strategies	x		8	
7. Data extraction	x		9	
8. Reported reliability	x		9	
9. Estimating the reliability induction and other sources of bias	x		9	
10. Data extraction of inducing studies	x		9	
11. Reliability of data extraction	x		9	
12. Transformation method			NR	
13. Statistical model	x		10	
14. Weighting method			NR	
15. Heterogeneity assessment	x		10	
16. Moderator analyses				NR
17. Additional analyses				NR
18. Software				NR
**RESULTS**	Yes	No	Page	NR
19. Results of the study selection process	x		11	
20. Mean reliability and heterogeneity	x		21	
21. Moderator analyses		x		
22. Sensitivity analyses		x		
23. Comparison of inducing and reporting studies		x		
24. Data set	x			
**DISCUSSION**	Yes	No	Page	NR
25. Summary of results			28	
26. Limitations	x		26	
27. Implications for practice	x		26	
28. Implications for future research	x		26	
**FUNDING**	Yes	No	Page	NR
29. Funding	x		28	
**PROTOCOL**	Yes	No	Page	NR
**30. Protocol**				x

Nota. NR = not reported. Source: Authors’ elaborations based on REGEMA checklist.

**Table A6:** PRISMA checklist.

Section and Topic	Item	Checklist	Location Where Item Is Reported
**Title**	1	Identify the report as a systematic review.	1
**Abstract**	2	See the PRISMA 2020 for Abstracts checklist.	2
**INTRODUCTION**			
**Rationale**	3	Describe the rationale for the review in the context of existing knowledge.	3
**Objectives**	4	Provide an explicit statement of the objective(s) or question(s) the review addresses.	7
**METHOD**			
**Eligibility criteria**	5	Specify the inclusion and exclusion criteria for the review and how studies were grouped for the syntheses	7
**Information** **sources**	6	Specify all databases, registers, websites, organisations, reference lists and other sources searched or consulted to identify studies. Specify the date when each source was last searched or consulted.	8
**Search strategy**	7	Present the full search strategies for all databases, registers and websites, including any filters and limits used.	8
**Selection process**	8	Specify the methods used to decide whether a study met the inclusion criteria of the review, including how many reviewers screened each record and each report retrieved, whether they worked independently, and if applicable, details of automation tools used in the process.	7
**Data collection** **process**	9	Specify the methods used to collect data from reports, including how many reviewers collected data from each report, whether they worked independently, any processes for obtaining or confirming data from study investigators, and if applicable, details of automation tools used in the process.	9
**Data items**	10a	List and define all outcomes for which data were sought. Specify whether all results that were compatible with each outcome domain in each study were sought (e.g., for all measures, time points, analyses), and if not, the methods used to decide which results to collect.	NR
	10b	List and define all other variables for which data were sought (e.g., participant and intervention characteristics, funding sources). Describe any assumptions made about any missing or unclear information.	9
**Study risk of bias assessment**	11	Specify the methods used to assess risk of bias in the included studies, including details of the tool(s) used, how many reviewers assessed each study and whether they worked independently, and if applicable, details of automation tools used in the process.	9
**Effect measures**	12	Specify for each outcome the effect measure(s) (e.g., risk ratio, mean difference) used in the synthesis or presentation of results.	NR
**Synthesis methods**	13a	Describe the processes used to decide which studies were eligible for each synthesis (e.g., tabulating the study intervention characteristics and comparing against the planned groups for each synthesis (item #5))	**NR**
	13b	Describe any methods required to prepare the data for presentation or synthesis, such as handling of missing summary statistics, or data conversions.	NR
	13c	Describe any methods used to tabulate or visually display results of individual studies and syntheses.	NR
	13d	Describe any methods used to synthesize results and provide a rationale for the choice(s). If meta-analysis was performed, describe the model(s), method(s) to identify the presence and extent of statistical heterogeneity, and software package(s) used.	9
	13e	Describe any methods used to explore possible causes of heterogeneity among study results (e.g., subgroup analysis, meta-regression)	10
	13f	Describe any sensitivity analyses conducted to assess robustness of the synthesized results	NR
**Reporting bias** **assessment**	14	Describe any methods used to assess risk of bias due to missing results in a synthesis (arising from reporting biases).	10
**Certainty** **assessment**	15	Describe any methods used to assess certainty (or confidence) in the body of evidence for an outcome.	10
**RESULTS**			
**Study selection**	16a	Describe the results of the search and selection process, from the number of records identified in the search to the number of studies included in the review, ideally using a flow diagram.	13
	16b	Cite studies that might appear to meet the inclusion criteria, but which were excluded, and explain why they were excluded.	7
**Study** **characteristics**	17	Cite each included study and present its characteristics.	NR
**Risk of bias in** **studies**	18	Present assessments of risk of bias for each included study.	NR
**Results of** **individual studies**	19	For all outcomes, present, for each study: (a) summary statistics for each group (where appropriate) and (b) an effect estimate and its precision (e.g., confidence/credible interval), ideally using structured tables or plots.	NR
**Results of** **syntheses**	20a	For each synthesis, briefly summarise the characteristics and risk of bias among contributing studies.	NR
	20b	Present results of all statistical syntheses conducted. If meta-analysis was done, present for each the summary estimate and its precision (e.g., confidence/credible interval) and measures of statistical heterogeneity. If comparing groups, describe the direction of the effect.	21
	20c	Present results of all investigations of possible causes of heterogeneity among study results.	21
	20d	Present results of all sensitivity analyses conducted to assess the robustness of the synthesized results.	NR
**Reporting biases**	21	Present assessments of risk of bias due to missing results (arising from reporting biases) for each synthesis assessed.	NR
**Certainty of** **evidence**	22	Present assessments of certainty (or confidence) in the body of evidence for each outcome assessed.	
**DISCUSSION**			
	23a	Provide a general interpretation of the results in the context of other evidence.	22
	23b	Discuss any limitations of the evidence included in the review.	26
	23c	Discuss any limitations of the review processes used.	26
	23d	Discuss implications of the results for practice, policy, and future research.	26
**OTHER INFORMATION**			
**Registration and protocol**	24a	Provide registration information for the review, including register name and registration number, or state that the review was not registered.	NR
	24b	Indicate where the review protocol can be accessed, or state that a protocol was not prepared.	NR
	24c	Describe and explain any amendments to information provided at registration or in the protocol.	NR
**Support**	25	Describe sources of financial or non-financial support for the review, and the role of the funders or sponsors in the review.	
**Competing interests**	26	Declare any competing interests of review authors.	
**Availability of** **data, code and** **other materials**	27	Report which of the following are publicly available and where they can be found: template data collection forms; data extracted from included studies; data used for all analyses; analytic code; any other materials used in the review.	NR

NA = Not Applicable. Source: Prepared by the authors on the basis of the PRISMA checklist.
